# Pyrolysis Kinetics and Thermodynamics of Ambient-Pressure-Dried Silica Aerogels Modified with Tri-, Di- and Mono-Methylsilyl Groups

**DOI:** 10.3390/gels12070594

**Published:** 2026-07-03

**Authors:** Xiaoxu Wu, Zhiyu Huo, Miao Liu, Qiao Wang, Yang Wang, Zhi Li

**Affiliations:** School of Resources and Safety Engineering, Central South University, Changsha 410083, China

**Keywords:** silica aerogels, pyrolysis kinetics, thermodynamics, reaction mechanism

## Abstract

Hydrophobic silica aerogels are widely used as thermal-insulation materials, but the thermal decomposition of their organic surface groups may affect their stability and safety during high-temperature service. In this study, ambient-pressure-dried silica aerogels modified with trimethylsilyl, dimethylsilyl, and methylsilyl groups were prepared and denoted as TSA, DSA, and MSA, respectively, to clarify how the degree of methyl substitution in the surface modifier controls the pyrolysis behavior of hydrophobic silica aerogels. Thermogravimetric analysis at different heating rates was combined with TG-FTIR, a model-free kinetic analysis, a model-fitting analysis and thermodynamic calculation. With decreasing methyl substitution from TSA to MSA, the aerogel framework became denser, the specific surface area decreased, and the contribution of solid-phase heat transfer increased slightly. The main pyrolysis process occurred at 250–800 °C and involved multiple overlapping reactions. The average activation energies of TSA, DSA, and MSA were 241.4, 246.6, and 285.5 kJ/mol according to the Kissinger–Akahira–Sunose (KAS) method and 243.0, 248.2, and 289.0 kJ/mol according to the Flynn–Wall–Ozawa (FWO) method, respectively. The higher activation energy of MSA indicates that the more condensed silica-rich framework and lower organic methyl content improves its resistance to the main degradation process. The model-fitting analysis further suggested an A1/2 mechanism for TSA and A2/5 mechanisms for DSA and MSA. TG-FTIR further confirmed the evolution of CO_2_, H_2_O, CH_4_, and C_2_H_4_ and revealed distinct gas-release behaviors among the three samples. These results demonstrate that the surface methyl-substitution structure governs the balance between hydrophobic modification, pore-structure preservation, pyrolysis resistance, and volatile-product release, providing a basis for selecting surface modifiers for thermally stable silica-aerogel insulation materials under oxygen-limited high-temperature conditions.

## 1. Introduction

In thermal-insulation applications, silica aerogels (SAs) are valued because their sol–gel-derived nanoporous networks can combine low density, high porosity, high specific surface area, and low thermal conductivity [1,2,3,4]. These structural characteristics make SAs promising candidates for thermal insulation, energy conservation, and building-safety-related applications [5,6,7,8]. In practical service environments, hydrophobic modification is usually required to improve moisture resistance and long-term stability, and hydrophobic silica aerogels (HSAs) generally exhibit better durability than hydrophilic silica aerogels [7]. However, the introduction of hydrophobic organosilicon groups also changes the thermal response of the silica framework. Methyl-containing surface groups may decompose, rearrange, or volatilize at elevated temperatures, thereby producing combustible or hazardous gaseous products and increasing the uncertainty of thermal safety during high-temperature exposure [9]. Therefore, the thermal behavior of HSAs is not determined only by the silica skeleton but also by the chemical structure and amount of the surface modifier. Trimethylsilyl, dimethylsilyl, and methylsilyl modifications differ in the number of methyl groups attached to silicon and in their possible contribution to surface capping, crosslinking, and network condensation. These differences may alter the pore structure, density, specific surface area, and thermal-degradation pathway of the final aerogels. Clarifying this structure–pyrolysis relationship is essential for selecting suitable hydrophobic modifiers for thermally stable aerogel insulation materials.

Previous studies [10,11,12,13] have demonstrated that the thermal behavior of HSAs is closely related to their surface modification chemistry. The multistep thermal decomposition, kinetic behavior, and reaction mechanisms of hydrophobic silica aerogels have been investigated under different atmospheres, and heat treatment has also been shown to affect the pore structure and pyrolysis behavior of silica aerogels. In addition, methylsilyl-modified silica aerogels have been reported to exhibit different combustion characteristics and thermal hazards, confirming that the type and amount of methylsilyl groups are important for thermal-safety evaluation. Li et al. investigated the pyrolysis behavior of silica aerogels modified with trimethylsilyl groups in an air atmosphere. Zhong et al. [14] investigated the thermal decomposition and conversion mechanisms of silica-aerogel-infused cork cells, providing further evidence that silica-aerogel-based materials can undergo complex thermal conversion processes involving both organic components and silica-derived porous structures. These studies provided useful information on the thermal degradation of hydrophobic aerogels, but the pyrolysis behavior under an inert atmosphere has not been fully clarified. However, most of these studies focused on a single type of hydrophobic modification or on the general pyrolysis behavior of hydrophobic silica aerogels, while the systematic relationship between methyl-substitution degree, aerogel structure, activation energy, apparent reaction model, and evolved-gas behavior remains insufficiently clarified. In particular, the effects of tri-, di-, and mono-methylsilyl groups on the decomposition stage, activation energy, reaction model, and volatile-product evolution still require systematic comparison. Such information is important for evaluating the intrinsic thermal hazards of HSAs independently of oxidation reactions.

Thermogravimetric analysis (TG) is an effective method for tracking mass loss and evaluating kinetic or thermodynamic parameters during thermal decomposition [15,16,17,18]. Nevertheless, TG results alone cannot identify the chemical composition of evolved gases. Fourier transform infrared spectroscopy (FTIR) can distinguish typical gas-phase products and functional groups according to their characteristic absorption bands, such as those related to H_2_O, CO_2_, CO, CH_4_, and other small molecules [19,20,21]. Therefore, coupling TG with FTIR enables the mass-loss process to be correlated with the release of gaseous products, which is particularly useful for analyzing the pyrolysis stages and volatile-generation behavior of organic–inorganic hybrid materials [19,20]. For HSAs with different methyl-substituted surface groups, this combined technique can provide direct evidence for identifying the gaseous products generated from the thermal decomposition of hydrophobic groups.

In this work, three ambient-pressure-dried HSAs modified with trimethylchlorosilane (TMCS), dimethyldichlorosilane (DMDCS), and methyltrichlorosilane (MTCS) were prepared and denoted as TSA, DSA, and MSA, respectively. These samples represent three methylsilyl surface-modification structures with decreasing methyl substitution from TSA to MSA. TG experiments at different heating rates were conducted under a nitrogen atmosphere to determine their pyrolysis characteristics. TG-FTIR was used to identify the gaseous products released during degradation. The Kissinger–Akahira–Sunose (KAS) and Flynn–Wall–Ozawa (FWO) methods were applied to evaluate the conversion-dependent activation energies, while the Malek and Coats–Redfern methods were used to analyze the apparent reaction models. By comparing TSA, DSA, and MSA, this study aims to clarify how the surface methyl-substitution structure affects the pore structure, pyrolysis kinetics, thermodynamic parameters, and volatile-product evolution of HSAs. The results are expected to provide a kinetic and mechanistic basis for selecting hydrophobic modifiers for silica aerogel insulation materials used under oxygen-limited or high-temperature conditions.

## 2. Results and Discussion

### 2.1. Basic Physicochemical Properties

**Figure 1:**
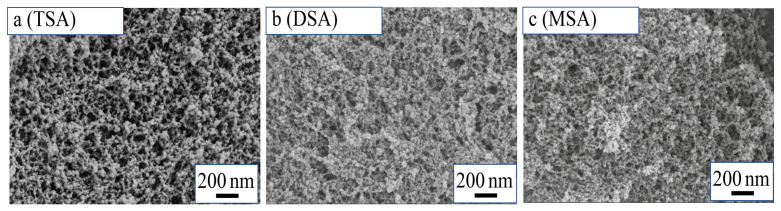
SEM images of (**a**) TSA, (**b**) DSA, and (**c**) MSA.

Figure 1 shows the SEM images of the three hydrophobic silica aerogels modified with different methylsilyl groups. All samples exhibited continuous three-dimensional nanoporous networks, indicating that the aerogel skeletons were generally preserved after ambient-pressure drying. However, clear differences in the pore morphology were observed among TSA, DSA, and MSA. TSA showed a relatively loose and homogeneous nanoporous network, whereas DSA and especially MSA displayed denser particle aggregation and less open pore structures. This morphological evolution suggests that the type of surface modifier not only determines the surface organic groups but also affects the development and preservation of the aerogel framework.

**Figure 2:**
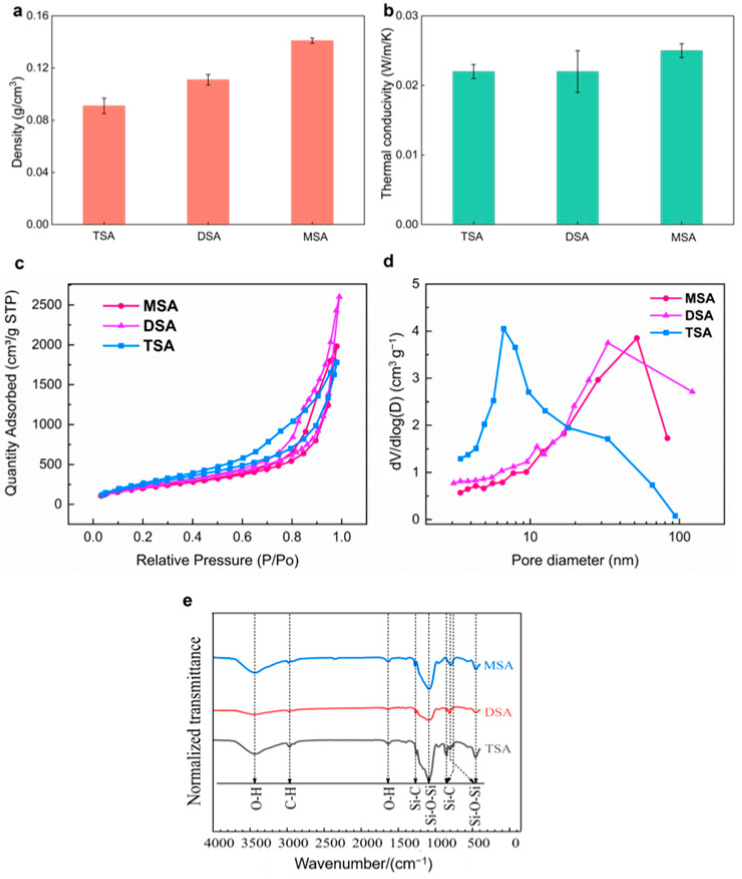
Basic physicochemical properties of TSA, DSA, and MSA: (**a**) density, (**b**) thermal conductivity, (**c**) N_2_ adsorption–desorption isotherms, (**d**) BJH pore-size distributions, and (**e**) FTIR spectra. The N_2_ adsorption–desorption isotherms and BJH pore-size distribution data in (**c**,**d**) were replotted from our previous study [22].

The structural differences observed by SEM are consistent with the density, thermal conductivity, and N_2_ physisorption results shown in Figure 2. The densities of TSA, DSA, and MSA were 0.091, 0.111, and 0.141 g/cm^3^, respectively, showing a gradual increase as the methyl substitution of the modifier decreased. As shown in Figure 2c, the N_2_ adsorption–desorption isotherms of all three aerogels exhibit typical mesoporous adsorption behavior, with a distinct increase in adsorbed volume at high relative pressures, indicating capillary condensation in the interconnected pore network. The corresponding BJH pore-size distributions further confirm the mesoporous characteristics of the aerogels (Figure 2d). These N_2_ physisorption data were replotted from our previous study for structural comparison. The previously reported BET specific surface areas decreased from 1002.4 m^2^/g for TSA to 877.7 m^2^/g for DSA and 773.3 m^2^/g for MSA [22]. This opposite trend between density and specific surface area indicates that the MSA sample possessed a more compact silica framework and reduced accessible pore volume. Such a denser network can also explain the slightly higher thermal conductivity of MSA. Although all three aerogels retained low thermal conductivity values, the increase from 0.022 W/(m·K) for TSA and DSA to 0.025 W/(m·K) for MSA indicates that the contribution of solid-phase heat transfer became more pronounced when the silica skeleton became more continuous.

The solid-state FTIR spectra further confirmed the successful introduction of methyl-containing organosilicon groups into the aerogel networks. The assignment of silica-related vibration bands was made by considering reported FTIR analyses of silica, silicate networks, and silica-based particles. The absorption band near 2978 cm^−1^ was attributed to C–H stretching vibrations [23], while the bands around 1275 and 849 cm^−1^ were assigned to Si–C-related vibrations in methylsilyl groups [24]. The bands near 800 and 452 cm^−1^ correspond to Si–O–Si vibrations [25], confirming the formation of a silica-based framework. The broad band around 3400 cm^−1^ and the band near 1630 cm^−1^ can be associated with adsorbed water and hydrogen-bonded O-H species. However, because KBr is hygroscopic, these bands should not be used to quantitatively compare the water content or humidity adsorption among the samples. Therefore, the FTIR analysis was mainly used to verify the presence of methylsilyl groups and the silica network rather than to draw conclusions on the relative moisture content of TSA, DSA, and MSA.

Overall, the structural characterization indicates that decreasing the methyl substitution from TSA to MSA resulted in a denser aerogel framework, lower specific surface area, and slightly higher thermal conductivity. The N_2_ adsorption–desorption isotherms and BJH pore-size distributions further demonstrate that the pore structures of the three aerogels are closely related to the methylsilyl modification type. These structural differences are important for interpreting the following thermal degradation behavior, because a denser silica-rich network may restrict volatile diffusion and increase the apparent resistance to pyrolysis.

### 2.2. Thermal Analysis

**Figure 3:**
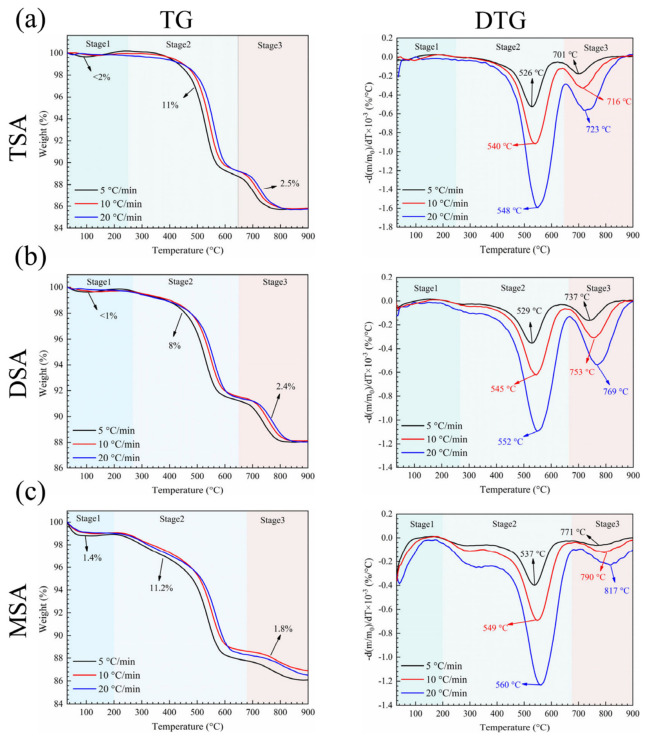
TG/DTG curves of (**a**) TSA, (**b**) DSA, and (**c**) MSA in nitrogen atmosphere at different heating rates.

Figure 3 presents the TG and DTG curves of TSA, DSA, and MSA obtained at heating rates of 5, 10, and 20 °C/min under a nitrogen atmosphere. The principal mass-loss region of the three aerogels was located between 250 °C and 800 °C. With an increasing heating rate, the characteristic temperatures shifted to higher values, which can be attributed to thermal lag and the limited time available for heat and mass transfer during dynamic heating.

The pyrolysis process can be divided into three main stages as shown in Table 1. In Stage 1, below approximately 250 °C, only slight mass loss was observed. This mass loss can mainly be attributed to physically adsorbed water and traces of residual pore liquids or volatile species from the preparation process, such as ethanol or n-hexane. The small mass loss in this stage indicates that most of the residual solvent had been removed during drying [26,27]. In Stage 2, from approximately 250 to 700 °C, the main mass loss occurred. This stage is associated with the decomposition and rearrangement of methyl-containing organosilicon groups, accompanied by the release of small-molecule volatile products. In Stage 3, from approximately 700 to 900 °C, further degradation of residual organic groups and structural rearrangement of the silica-rich network occurred. The release of H_2_O in this high-temperature region can be partly related to condensation reactions between residual silanol or hydroxyl-containing sites, rather than simple evaporation of physically adsorbed water [16,28].

Although the three samples showed similar multistep degradation characteristics, their DTG profiles and characteristic temperatures were different. TSA and DSA, which contained more methyl groups in their surface-modification structures, exhibited more pronounced mass loss in the main degradation region. This behavior suggests that a higher organic-group content provides more decomposable methyl-containing units. In contrast, MSA showed denser morphology, higher density, and lower BET specific surface area, which indicates a more compact silica-rich framework. Such a structure may delay the transport of volatile products and increase the apparent resistance to thermal degradation. Therefore, the difference in TG/DTG behavior among TSA, DSA, and MSA should be interpreted as the combined effect of surface organic-group content and aerogel network density.

The above results indicate that the type of methylsilyl modifier affects not only the amount of decomposable organic groups but also the structural compactness of the aerogel skeleton. This dual effect provides the structural basis for the differences in activation energy and evolved-gas behavior discussed in the following sections.

**Table 1:** Pyrolysis characteristics of HSAs at different heating rates.

Samples	*β* (°C/min)	Stage 1		Stage 2			Stage 3			Residue at 900 °C (%)
		T_o_ (°C)	T_e_ (°C)	T_o_ (°C)	T_p_ (°C)	T_e_ (°C)	T_o_ (°C)	T_p_ (°C)	T_e_ (°C)	
TSA	5	31	226	226	526	618	618	701	900	85.5
	10	31	245	245	540	635	635	716	900	85.6
	20	31	266	266	548	652	652	723	900	85.6
DSA	5	31	235	235	529	645	645	737	900	88.1
	10	31	267	267	545	652	652	753	900	88.2
	20	31	274	274	552	666	666	769	900	88.1
MSA	5	31	183	183	537	657	657	771	900	86.0
	10	31	199	199	549	678	678	790	900	86.8
	20	31	204	204	560	688	688	817	900	86.4

### 2.3. Kinetic Parameters Based on Model-Free Methods

**Figure 4:**
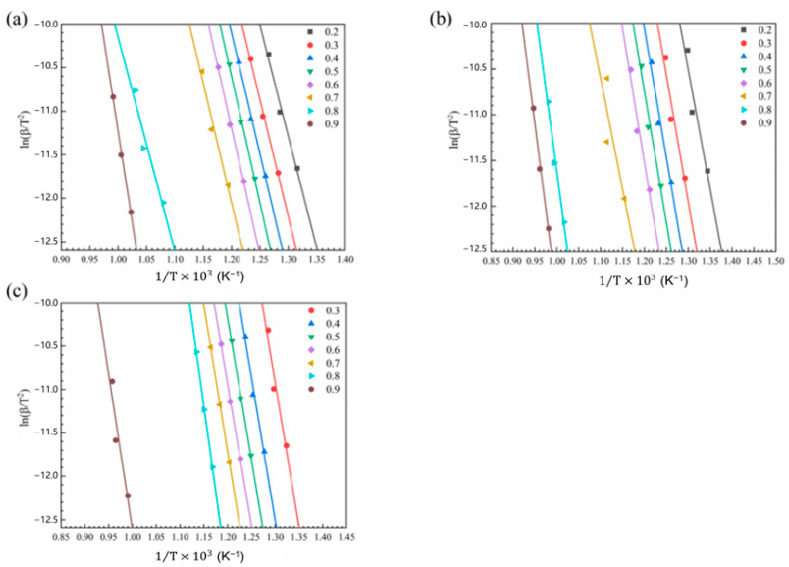
KAS plots for different conversion rates: (**a**) TSA, (**b**) DSA, and (**c**) MSA.

**Figure 5:**
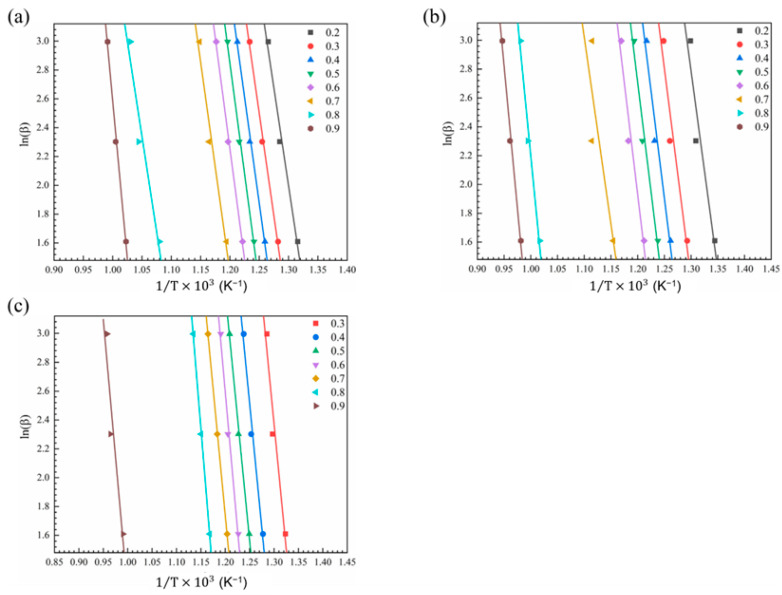
FWO plots for different conversion rates: (**a**) TSA, (**b**) DSA, and (**c**) MSA.

**Figure 6:**
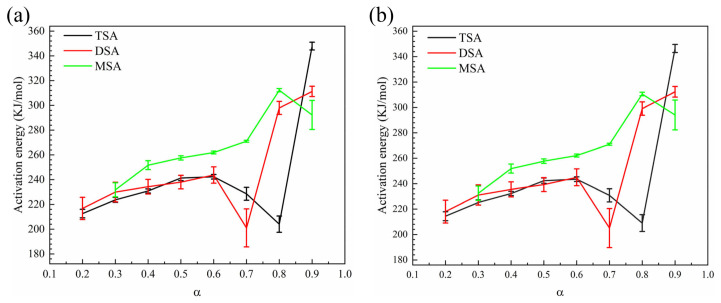
Changes in the activation energies during the pyrolysis of HSAs derived from (**a**) KAS and (**b**) FWO methods.

Based on the model-free KAS and FWO methods, a conversion degree (α) was selected in the range of 0.2–0.9 for TSA and DSA and 0.3–0.9 for MSA. The corresponding linear fitting plots obtained from the KAS and FWO methods are presented in Figure 4 and Figure 5, respectively. The apparent activation energy (Ea) at each conversion level was calculated from the slope of the fitted line, and the resulting kinetic parameters are summarized in Table 2. The conversion-dependent Ea profiles derived from the two methods are compared in Figure 6.

The Ea values obtained from the KAS and FWO methods showed similar magnitudes and variation trends, indicating that the two model-free methods gave consistent kinetic results. The dependence of Ea on α suggests that the pyrolysis of hydrophobic silica aerogels is not governed by a single elementary reaction but involves several overlapping processes, including the release of weakly bound species, decomposition of methyl-containing organosilicon groups, volatile-product formation, mass transfer through the porous framework, and rearrangement of the silica-rich skeleton [29].

For TSA, the Ea values increased from 212.6 to 242.3 kJ/mol in the range of α = 0.2–0.6 according to the KAS method, and a similar trend was obtained by the FWO method. This increase corresponds to the main degradation region observed in the TG/DTG curves, where relatively labile methyl-containing silyl groups started to decompose. At higher conversion levels, the Ea values fluctuated rather than increasing monotonically. This behavior indicates that the degradation of TSA involved overlapping reactions instead of a continuous single-step process. The sharp increase in Ea at α = 0.9 suggests that the remaining organic groups or more thermally resistant organosilicon structures required higher energy for further degradation [30].

DSA showed kinetic behavior like that of TSA in the low- and medium-conversion ranges. The Ea values increased from 216.7 to 243.7 kJ/mol at α = 0.2–0.6 by the KAS method, indicating that the initial and main degradation stages were also dominated by the decomposition of methyl-containing surface groups. However, the fitting reliability at α = 0.7 was relatively poor, as reflected by the low R^2^ values in both the KAS and FWO results. Therefore, the Ea value at this conversion should not be overinterpreted as an independent mechanistic transition. Instead, it more likely reflects the strong overlap between the end of the main methyl-group decomposition stage and the beginning of subsequent residual-structure degradation. At higher conversions, the Ea values increased again, suggesting that the remaining organosilicon structures became more resistant to thermal decomposition.

MSA exhibited the highest average Ea among the three aerogels. The average activation energies of MSA were 285.5 kJ/mol by KAS and 289.0 kJ/mol by FWO, which were distinctly higher than those of TSA and DSA. This result indicates that the mono-methylsilyl-modified aerogel had the highest apparent resistance to the main pyrolysis process. This behavior can be attributed to both its chemical structure and physical morphology. Compared with TSA and DSA, MSA contained fewer methyl groups per silicon center, reducing the contribution of readily decomposable organic units. In addition, MSA had the highest density and the lowest BET specific surface area, indicating a denser silica-rich framework. Such a compact network can restrict volatile diffusion and increase the apparent energy barrier for degradation, thereby leading to higher Ea values over most of the conversion range.

The decrease in Ea for MSA from α = 0.8 to 0.9 requires further consideration. This decrease does not mean that the initial MSA structure had poor thermal stability. Rather, it suggests that the major decomposable methyl-containing structures had already been largely consumed before the final conversion stage. Consequently, the later stage was less dominated by Si-CH_3_ decomposition and more controlled by residual structural rearrangement, limited volatile release, and condensation or reorganization of the silica-rich framework. This interpretation is consistent with the TG/DTG results, where MSA showed a more compact structure and delayed high-temperature degradation behavior.

Overall, the model-free kinetic analysis demonstrates that the surface modifier structure strongly affects the apparent pyrolysis resistance of the aerogels. The average Ea followed the order MSA > DSA ≈ TSA, showing that decreasing the methyl substitution from trimethylsilyl to methylsilyl modification increased the apparent energy requirement for the main pyrolysis process. This trend is closely related to the denser silica-rich network, lower BET specific surface area, and reduced content of readily decomposable methyl groups in MSA. Therefore, the kinetic results provide direct evidence that the pyrolysis behavior of hydrophobic silica aerogels is jointly controlled by surface organic-group content and framework compactness.

### 2.4. Reaction Mechanism Functions

**Figure 7:**
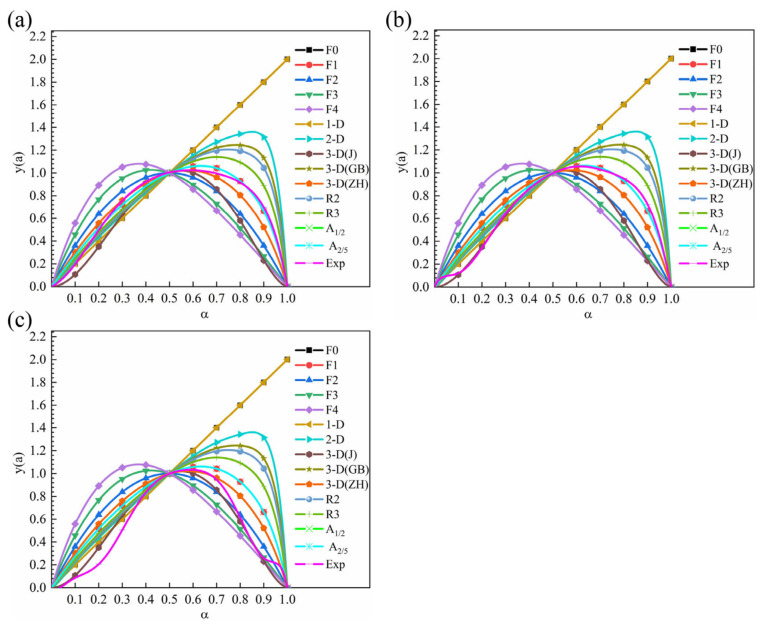
*y*(*α*) versus conversion (*α*) master plots of (**a**) TSA, (**b**) DSA and (**c**) MSA at 10 °C/min.

The reaction mechanism functions of HSA pyrolysis were further evaluated using the Malek master-plot method and the Coats–Redfern method. A heating rate of 10 °C/min was selected as the representative condition for comparing the experimental and theoretical master plots, so as to reduce the possible influence of heating-rate variation on model discrimination [31]. The experimental and theoretical y(*α*) curves are presented in Figure 7. For TSA and DSA, the experimental y(α) curves were closer to the nucleation-growth-type models than to the reaction-order or diffusion models. This result suggests that the pyrolysis of TSA and DSA may involve the formation of newly decomposed regions within the solid matrix, followed by the growth of these regions as the decomposition proceeds [32]. In this context, the A-type models should be understood as apparent solid-state reaction models rather than elementary molecular reactions. The A1/2 and A2/5 functions describe nucleation-and-growth-controlled conversion behavior with different apparent growth exponents, reflecting differences in the formation and propagation of reactive regions during thermal degradation.

The MSA sample showed different behavior in the Malek master plot. Its experimental curve was closer to the three-dimensional diffusion model 3-D (ZH), suggesting that diffusion-related resistance contributed more strongly to the pyrolysis of MSA [33]. This result is consistent with the structural characterization, where MSA exhibited the highest density and the lowest BET specific surface area. The more compact silica-rich framework of MSA may hinder the outward diffusion of volatile products and promote the formation of a residual product layer during the later stage of degradation. Therefore, the Malek analysis indicates that the pyrolysis of MSA cannot be described simply by the same nucleation-growth behavior observed for TSA and DSA.

**Figure 8:**
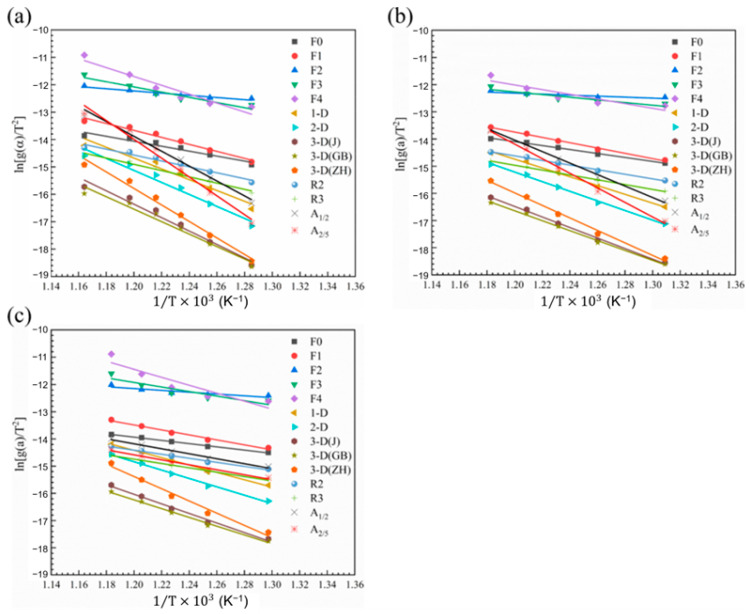
The CR curves of three hydrophobic SAs, (**a**) TSA, (**b**) DSA, and (**c**) MSA, at a heating rate of 10 °C/min.

To further determine the most probable reaction mechanism, the Coats–Redfern method was applied in parallel with the Malek analysis. Figure 8 presents the plots of ln(g(*α*)/T^2^) versus 1/T for different solid-state reaction models at a heating rate of 10 °C/min. The activation energies calculated from the slopes of the fitted lines are summarized in Table 3, Table 4 and Table 5 for TSA, DSA, and MSA, respectively. The candidate mechanism whose average activation energy at the three heating rates was closest to the values obtained from the KAS and FWO methods was considered the most suitable model for describing the pyrolysis process [34].

For TSA, the average Ea calculated using the A1/2 model was approximately 238.1 kJ/mol, which was close to the average Ea values obtained by the KAS method (241.4 kJ/mol) and the FWO method (243.0 kJ/mol). Therefore, A1/2 was selected as the apparent reaction model for TSA. This result indicates that the pyrolysis of TSA was mainly associated with random nucleation and subsequent growth of decomposition regions, which is reasonable considering the relatively loose porous network and higher content of methyl-containing surface groups in TSA.

For DSA, the Ea value obtained using the A2/5 model was closer to those obtained by the KAS and FWO methods than the values calculated from the other models. Therefore, A2/5 was selected as the apparent reaction model for DSA. Compared with TSA, DSA contained fewer methyl groups per silicon center and possessed a relatively denser framework. These differences may change the propagation behavior of the decomposed regions during pyrolysis, leading to a different apparent nucleation-growth exponent.

For MSA, the CR analysis also suggested A2/5 as the closest apparent model when the criterion of activation-energy agreement was used. However, this result should not be interpreted as evidence that MSA pyrolysis is governed only by nucleation and growth. The Malek master plot showed stronger agreement with the 3-D diffusion model, indicating that diffusion resistance also played an important role. Therefore, the pyrolysis of MSA is better described as a mixed process: the early and middle stages may involve structural transformation and growth of decomposed regions, whereas the later stage is increasingly affected by volatile diffusion through the denser silica-rich framework and residual product layer. In this sense, A2/5 represents the overall apparent model obtained from CR fitting, while the Malek analysis reveals the diffusion-related contribution in MSA.

Overall, the model-fitting results demonstrate that the pyrolysis of HSAs with different methylsilyl surface structures cannot be reduced to a single elementary reaction. TSA and DSA mainly exhibited nucleation-growth-controlled apparent behavior, whereas MSA showed mixed kinetic features involving both apparent nucleation-growth behavior and diffusion-related resistance. This distinction is consistent with the structural and kinetic results: decreasing methyl substitution from TSA to MSA produced a denser silica-rich framework, which increased the apparent activation energy and enhanced diffusion limitations during the later degradation stage.

### 2.5. TG-FTIR Analysis of Evolved Gases

TG-FTIR was used to identify the gaseous products released during the pyrolysis of TSA, DSA, and MSA and to correlate the gas-evolution behavior with the TG/DTG degradation stages. The TG-FTIR measurements were conducted under a helium atmosphere at a heating rate of 10 °C/min. The main evolved gases identified from the FTIR spectra were H_2_O, CO_2_, CH_4_, and C_2_H_4_, as summarized in Table 6. These gases were assigned according to their characteristic gas-phase infrared absorption bands [19,21,35]. The H_2_O-related bands were mainly associated with O–H stretching and bending vibrations, while CO_2_ was identified from its characteristic gas-phase asymmetric stretching absorption. The hydrocarbon products, including CH_4_ and C_2_H_4_, were assigned according to their C–H-related vibration bands. Because gas-phase H_2_O and CO_2_ absorptions are susceptible to residual atmospheric H_2_O/CO_2_ in the optical path and gas cell, their evolution profiles were interpreted together with the corresponding TG/DTG curves rather than independently [36,37]. In addition, irregular CO_2_ and H_2_O absorption signals appeared during the whole pyrolysis process, which may be related to background interference or ghost peaks caused by an imbalance in the optical path during testing.

**Figure 9:**
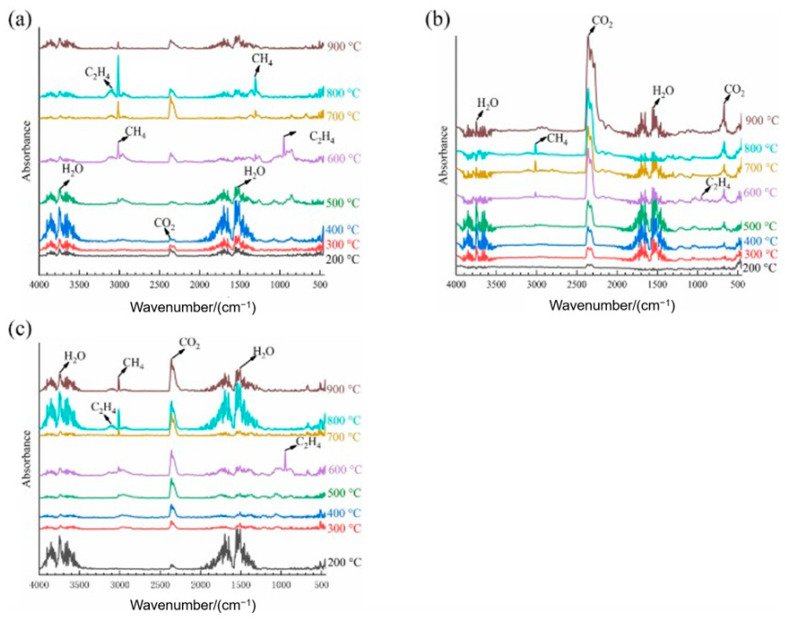
Temperature-dependent FTIR absorbance profiles of the main evolved gases released from (**a**) TSA, (**b**) DSA, and (**c**) MSA during pyrolysis at 10 °C/min under a helium atmosphere.

Figure 9 shows the temperature-dependent FTIR absorbance profiles of the main gaseous products during the pyrolysis of the three aerogels. For TSA, the gas-evolution behavior was closely related to the decomposition of trimethylsilyl surface groups. A weak H_2_O signal appeared at the low-temperature stage, which can be mainly attributed to the removal of physically adsorbed water and weakly bound hydroxyl-containing species. In the main degradation region, CH_4_ and C_2_H_4_ were detected, indicating that methyl-containing organosilicon groups participated in the pyrolysis process. The formation of these hydrocarbon products can be associated with the cleavage of Si–CH_3_ bonds, followed by hydrogen abstraction, recombination, or dehydrogenation reactions of methyl-related fragments [19,20,38,39]. Therefore, the TG-FTIR results support the assignment of the 250–700 °C region to the main decomposition of organic surface groups. At higher temperatures, the H_2_O signal can be partly attributed to condensation reactions between residual hydroxyl-containing sites in the silica-rich framework rather than simple evaporation of physically adsorbed water.

DSA released similar types of gaseous products, but the relative evolution behavior differed from that of TSA. The detection of CH_4_ and C_2_H_4_ confirms that dimethylsilyl-derived groups also decomposed during the main pyrolysis stage. Compared with TSA, DSA contained fewer methyl groups per silicon center and exhibited a relatively denser aerogel framework. These structural differences may reduce the amount of readily releasable hydrocarbon fragments and shift part of the gas evolution to a higher temperature region. Thus, DSA showed intermediate behavior between the methyl-rich TSA and the more silica-rich MSA. This observation is consistent with the TG/DTG and kinetic results, indicating that the modifier structure affects both the decomposition temperature range and the apparent activation energy.

MSA exhibited a more distinct gas-evolution behavior. CH_4_, C_2_H_4_, CO_2_, and H_2_O were detected over a relatively broad temperature range, indicating that its pyrolysis involved not only methyl-group decomposition but also high-temperature structural rearrangement of the silica-rich network. Because MSA had the highest density and the lowest BET specific surface area, the diffusion of volatile products through the compact framework may have been more restricted. In addition, the lower methyl content of MSA reduced the contribution of readily decomposable organic groups, while the more condensed silica-rich structure promoted high-temperature network rearrangement and condensation reactions. This can explain the broader gas-release process and the more evident high-temperature H_2_O evolution observed for MSA.

The TG-FTIR results are consistent with the model-free kinetic analysis. TSA and DSA, which contained more methyl groups in their surface-modification structures, generated hydrocarbon products mainly through the decomposition of methyl-containing organosilicon groups. In contrast, MSA showed higher Ea and broader gas-evolution behavior because of its denser silica-rich framework and lower content of readily decomposable methyl groups. These findings demonstrate that the surface methyl-substitution structure controls not only the pyrolysis resistance of hydrophobic silica aerogels but also the type and release behavior of volatile products. The release of hydrocarbon gases from methyl-containing surface groups is also consistent with previous thermal-hazard and pyrolysis studies of methylsilyl-modified silica aerogels [10,13]. It should be noted that TMCS, DMDCS, and MTCS used in this study do not contain Si–H bonds; therefore, Si–H-related absorption was not expected in the solid FTIR spectra.

### 2.6. Thermodynamic Analysis

**Figure 10:**
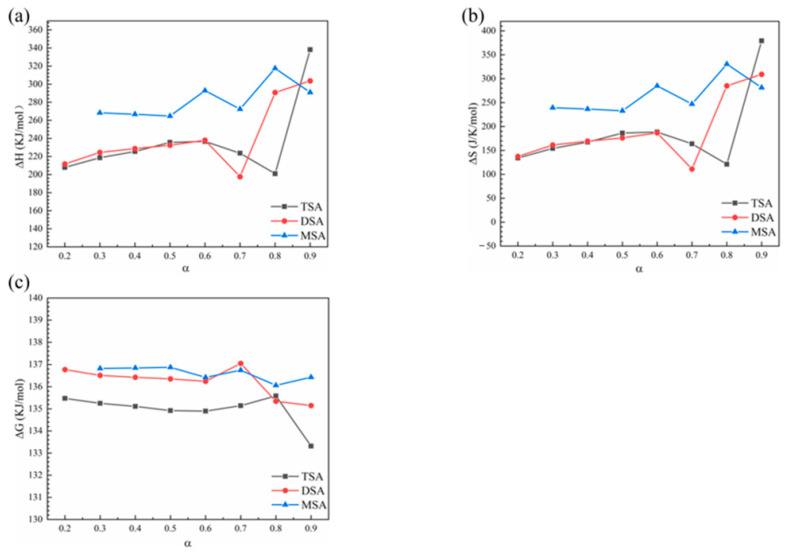
Thermodynamic profiles calculated for TSA, DSA, and MSA at 10 °C/min: (**a**) *ΔH*, (**b**) *ΔS*, and (**c**) *ΔG*.

To further evaluate the energetic characteristics of HSA pyrolysis, the thermodynamic parameters of TSA, DSA, and MSA were calculated from the apparent activation energies obtained by the KAS and FWO methods. The heating rate of 10 °C/min was selected for this calculation because it provided representative kinetic information and reduced the possible influence of heating-rate-dependent deviations [38,40,41]. The calculated pre-exponential factor (A), enthalpy change (ΔH), Gibbs free energy change (ΔG), and entropy change (ΔS) are presented in Figure 10 and Table 7.

The lnA values varied with conversion for all three aerogels, confirming that their pyrolysis did not proceed through a single elementary reaction. In general, low A values are usually associated with surface-controlled processes, whereas high A values are related to more complex reaction events involving bond cleavage, structural rearrangement, and volatile release [42]. In this study, the relatively high lnA values and their conversion-dependent variation indicate that the pyrolysis of HSAs involved multiple overlapping processes, which is consistent with the TG/DTG, TG-FTIR, and model-free kinetic results.

The ΔH values show a trend like the apparent activation energies. MSA exhibited generally higher ΔH values than TSA and DSA over most of the conversion range, indicating that more energy was required to drive the main degradation process of the mono-methylsilyl-modified aerogel. This result agrees with the higher Ea values of MSA obtained from the KAS and FWO methods. The higher ΔH of MSA can be attributed to its denser silica-rich framework, lower BET specific surface area, and lower content of readily decomposable methyl groups. These structural features increase the energy demand for organic-group decomposition, volatile diffusion, and framework rearrangement. Previous thermodynamic analyses have suggested that the difference between Ea and ΔH can be used to evaluate the energy requirement for forming activated complexes during thermal degradation [43,44]. In the present study, the relatively large energy demand further supports that the pyrolysis of HSAs, especially MSA, is not a facile decomposition process.

The ΔG values changed only slightly among the three samples and across the conversion range investigated. This limited variation indicates that the free-energy requirement for forming activated complexes remained relatively stable during the main pyrolysis process. Therefore, the differences in pyrolysis behavior among TSA, DSA, and MSA were not mainly caused by a large fluctuation in ΔG. Instead, they were more closely associated with differences in ΔH, framework compactness, methyl-group content, and mass-transfer resistance. This interpretation is consistent with the structural characterization and kinetic analysis, where MSA showed the highest density, the lowest specific surface area, and the highest average activation energy.

The ΔS values of the three samples were positive throughout the examined conversion range. This indicates that the pyrolysis process was accompanied by an increase in system disorder. Such an increase can be attributed to the generation and release of gaseous products, cleavage of methyl-containing organosilicon groups, and progressive rearrangement of the silica-based framework. Although condensation and residue formation may locally reduce disorder, the overall positive ΔS values suggest that volatile formation and structural transformation were dominant during the pyrolysis process [18].

Overall, the thermodynamic results support the kinetic and TG-FTIR analyses. The higher ΔH and Ea values of MSA demonstrate their stronger resistance to the main pyrolysis process, while the relatively stable ΔG values indicate that the formation of activated complexes required a comparable free-energy level among the three aerogels. The positive ΔS values further confirm that pyrolysis involved volatile release and structural reorganization. These results show that the thermodynamic behavior of HSAs is strongly affected by the surface methyl-substitution structure and the compactness of the silica-rich framework.

## 3. Conclusions

In this study, three ambient-pressure-dried hydrophobic silica aerogels modified with trimethylsilyl, dimethylsilyl, and methylsilyl groups were comparatively investigated to clarify the effect of surface methyl-substitution structure on pyrolysis behavior under a nitrogen atmosphere. The results show that the type of methylsilyl modifier affected not only the surface organic groups but also the pore structure and framework compactness of the aerogels. With decreasing methyl substitution from TSA to MSA, the aerogel framework became denser, the BET specific surface area decreased, and the thermal conductivity increased slightly, indicating a stronger contribution from the solid silica skeleton.

TG/DTG analysis showed that all three aerogels underwent a multistep pyrolysis process, mainly occurring between 250 and 800 °C. The main mass-loss stage was associated with the decomposition and rearrangement of methyl-containing organosilicon groups, while the high-temperature stage was related to further degradation of residual organic groups and structural rearrangement or condensation of the silica-rich framework. TSA and DSA, which contained more methyl groups in their surface-modification structures, exhibited more evident degradation associated with methyl-containing units. In contrast, MSA showed a more compact silica-rich framework, which increased the apparent resistance to the main pyrolysis process.

The model-free kinetic results obtained by the Kissinger–Akahira–Sunose (KAS) and Flynn–Wall–Ozawa (FWO) methods showed consistent trends. The average activation energies of TSA, DSA, and MSA were 241.4, 246.6, and 285.5 kJ/mol by KAS and 243.0, 248.2, and 289.0 kJ/mol by FWO, respectively. The higher activation energy of MSA indicates that the mono-methylsilyl-modified aerogel had the highest apparent resistance to pyrolysis, which can be attributed to its denser silica-rich network and lower content of readily decomposable methyl groups. The decrease in activation energy at high conversion for MSA suggests that most decomposable organic groups had already been consumed and that the later stage was mainly governed by residual structural rearrangement and limited volatile release.

The model-fitting analysis indicated that the pyrolysis behavior of the aerogels could be described by apparent solid-state reaction models rather than a single elementary reaction. TSA was mainly associated with an A1/2-type nucleation-growth model, while DSA and MSA were better described by A2/5-type apparent models. For MSA, the denser framework may also introduce diffusion-related resistance during the later degradation stage, suggesting that its pyrolysis involves mixed kinetic features.

TG-FTIR analysis confirmed the release of H_2_O, CO_2_, CH_4_, and C_2_H_4_ during pyrolysis. The hydrocarbon products were mainly derived from the decomposition of methyl-containing surface groups, whereas the high-temperature H_2_O release was associated with condensation reactions of residual hydroxyl-containing sites in the silica-rich framework. The different gas-evolution profiles of TSA, DSA, and MSA demonstrate that the surface methyl-substitution structure controls both volatile-product formation and pyrolysis resistance. Overall, this study shows that reducing the number of methyl groups in the surface modifier can enhance the apparent pyrolysis resistance of silica aerogels, but it also leads to a denser framework and slightly increased thermal conductivity. These findings provide a basis for selecting surface modifiers for hydrophobic silica aerogel insulation materials used under oxygen-limited or inert high-temperature conditions.

## 4. Materials and Methods

### 4.1. Raw Materials

Tetraethoxysilane (TEOS, 98%) was selected as the silica precursor. Ethanol (EtOH, 99.7%) and n-hexane were used as the solvent and solvent-exchange medium, respectively, while hydrochloric acid (HCl, 36.5%) and an ammonia solution (NH_3_·H_2_O, 26.5–28.5%) served as the acid and base catalysts. These chemicals were supplied by Sinopharm Chemical Reagent Co., Ltd. (Shanghai, China). Trimethylchlorosilane (TMCS, 98%), dimethyldichlorosilane (DMDCS, 98%), and methyltrichlorosilane (MTCS, 99%) were used for surface modification and were obtained from Shanghai Aladdin Biochemical Technology Co., Ltd. (Shanghai, China).

### 4.2. Sample Preparation

The hydrophobic silica aerogels were prepared by a sol–gel process followed by solvent exchange, surface modification, and ambient-pressure drying, according to our previously reported method with slight modifications [45]. Briefly, tetraethoxysilane (TEOS) was used as the silica precursor, ethanol was used as the solvent, and hydrochloric acid and the ammonia solution were used as the acid and base catalysts, respectively. TEOS was first mixed with ethanol, deionized water, and hydrochloric acid to form an acidic sol, which was hydrolyzed in a water bath at 45 °C for 12 h. The ammonia solution was then added to promote gelation, and the obtained wet gels were aged at 45 °C for 3 h.

After gelation and aging, ethanol was added to replace the water in the wet gels. The ethanol exchange was performed for 24 h, and the ethanol was refreshed every 12 h. Subsequently, n-hexane was used to replace ethanol in the gels under the same solvent-exchange procedure. The n-hexane-exchanged wet gels were then subjected to surface modification using trimethylchlorosilane (TMCS), dimethyldichlorosilane (DMDCS), or methyltrichlorosilane (MTCS) in the n-hexane solution for 12 h. The aerogels modified with TMCS, DMDCS, and MTCS were denoted as TSA, DSA, and MSA, respectively.

After surface modification, the gels were dried under ambient pressure to obtain hydrophobic silica aerogels. To remove free moisture before the thermal analysis, the dried samples were further treated in an oven at 120 °C for 2 h. Finally, all samples were ground and sieved to 100 mesh before TG and TG-FTIR measurements to minimize the influence of particle-size differences on the thermal analysis.

### 4.3. Characterization

The microstructure of the aerogel samples was examined using a field-emission scanning electron microscope (FE-SEM, ZEISS Sigma 300, Oberkochen, Germany). Before observation, the samples were fixed on conductive adhesive tape and coated with a thin conductive layer to reduce charging during electron-beam irradiation.

The apparent density of the aerogels was determined using a tap density tester (ZS-202, Liaoning Instrument Research Institute, Dandong, China). For each measurement, the powdered sample was placed into a 10 mL graduated cylinder and vibrated at 300 rpm for 10 min. The density was calculated from the measured mass and the stabilized volume after tapping. Each sample was measured repeatedly, and the average value was used for comparison.

Nitrogen adsorption–desorption measurements were carried out at 77 K using an automatic surface area and porosity analyzer (AUTOSORB IQ, Quantachrome, Boynton Beach, FL, USA). Before testing, the samples were degassed to remove physically adsorbed species. The specific surface area was calculated using the Brunauer–Emmett–Teller (BET) method, and the pore-size distribution was obtained from the desorption branch using the Barrett–Joyner–Halenda (BJH) method.

The room-temperature thermal conductivity of the aerogels was measured by the transient hot-wire method using a thermal conductivity analyzer (TC3000E, XIATECH, London, England The samples were gently filled into the sample holder to avoid excessive compression, and the measurements were performed under ambient conditions.

The chemical structure of the solid aerogels was characterized using Fourier transform infrared spectroscopy (FTIR, Nicolet 8700, Thermo Fisher/Nicolet, Madison, WI, USA). The spectra were collected over the wavenumber range of 4000–400 cm^−1^. For the KBr pellet measurements, the aerogel powders were mixed with dried KBr and pressed into transparent pellets before analysis. The obtained spectra were used to identify the Si–O–Si framework and methylsilyl-related surface groups. TG-FTIR was performed under a helium carrier gas.

Thermogravimetric analysis (TG) was performed using a thermal analyzer (SDT Q600, Waters, Milford, MA, USA) at heating rates of 5, 10, and 20 °C/min under a nitrogen atmosphere. TG-FTIR analysis was conducted using a thermogravimetric analyzer coupled with a Fourier transform infrared spectrometer (PerkinElmer TGA8000-FTIR-GCMS-ATD, Waltham, MA, USA). The samples were heated from 30 to 1000 °C, and the evolved gases were transferred to the FTIR gas cell for online analysis. The gas-phase FTIR spectra were recorded in the range of 4000–450 cm^−1^ with a resolution of 4 cm^−1^.

### 4.4. Kinetic Methods and Thermodynamic Analysis

The reaction rate can be expressed as a function of temperature and conversion by the Arrhenius equation:

(1) $$\frac{d\alpha }{dt}=Ae^{-(E_{a}/RT)}f(\alpha )$$

where *A*, *E_a_* and f(α) refer to the pre-exponential factor, the apparent activation energy and the differential form of reaction mechanism function, respectively. *R* is the gas constant, *T* is the reaction temperature, and *α* refers to conversion degree which can be expressed as follows:

(2) $$\alpha =\frac{m_{0}-m_{t}}{m_{0}-m_{\infty }}$$

where $m_{0}$, $m_{\infty }$ and $m_{t}$ refer to the mass of the initial samples, final samples and samples at time *t*, respectively.

There is a linear relationship between reaction temperature and time, $T=T_{0}+\beta t$, and the pyrolysis reaction rate can be expressed as follows:

(3) $$\frac{d\alpha }{dT}=\frac{A}{\beta }e^{-(E_{a}/RT)}f(\alpha )$$

Two model-free methods, KAS and FWO, were chosen to study the kinetic analysis of the pyrolysis of the HSAs, and the expressions are as follows respectively [38,46,47]:

(4) $$KAS:ln(\frac{\beta }{T^{2}})=ln(\frac{AR}{E_{a}g(\alpha )})-\frac{E_{a}}{RT}$$

(5) $$FWO:ln\beta =ln(\frac{AE_{a}}{Rg(\alpha )})-5.331-1.052\frac{E_{a}}{RT}$$

There is a linear relationship between $\frac{1}{T}$, $ln(\frac{\beta }{T^{2}})$ (KAS), and lnβ (FWO) at different heating rates. *E_a_* is obtained by the slope of the line.

Model-fitting methods are usually used to determine the reaction mechanism function. In this study, the method of combining Malek and Coats–Redfern (CR) was used to increase the accuracy of the calculation results [48,49].

The Malek method was used to identify the most probable kinetic model by comparing the normalized experimental master plot with the corresponding theoretical curves for different solid-state reaction mechanisms [50]. The common pyrolysis mechanism functions are summarized in Table 8. And the expression for *y*(*α*) is defined as follows:

(6) $$y(\alpha )=\frac{f(\alpha )\cdot G(\alpha )}{f(0.5)\cdot G(0.5)}={\left(\frac{T}{T_{0.5}}\right)}^{2}\frac{\left(\frac{d\alpha }{dt}\right)}{{\left(\frac{d\alpha }{dt}\right)}_{0.5}}$$

where the left side of the equation represents the theoretical curve, and the right side represents the experimental curve, and $T_{0.5}$ and ${\left(\frac{d\alpha }{dt}\right)}_{0.5}$ refer to the temperature and reaction rate at α=0.5, respectively.

The CR method expression is obtained by introducing $2RT/E_{a}\to 0$ into Equation (3) [51], and *E_a_* is obtained by the slope of the line:

(7) $$ln\frac{g(\alpha )}{T^{2}}=ln(\frac{AR}{\beta E_{a}})-\frac{E_{a}}{RT}$$

To further characterize the energetic features of HSA pyrolysis, the pre-exponential factor (*A*), enthalpy change (Δ*H*), Gibbs free energy change (Δ*G*), and entropy change (Δ*S*) were calculated using the following equations:

(8) $$A=\beta E_{a}\exp(\frac{E_{a}}{RT_{m}})/(RT_{m}^{2})$$

(9) $$\Delta H=E_{a}-RT_{\alpha }$$

(10) $$\Delta G=E_{a}+RT_{m}\ln(\frac{k_{B}T_{m}}{hA})$$

(11) $$\Delta S=\frac{\Delta H-\Delta G}{T_{m}}$$

where $T_{m}$ and $T_{\alpha }$ are the temperature of the peak value of the DTG curve and conversion rate, respectively; $k_{B}$ is the Boltzmann constant, $k_{B}=1.381\times 10^{-23}$ J/K; and h is the Planck constant, $h=6.626\times 10^{-34}$ J·s.

## Figures and Tables

**Table 2:** Calculation results of *E_a_* by the KAS and FWO method.

Samples	KAS Method			FWO Method		
	α	*E_a_* (kJ/mol)	R^2^	α	*E_a_* (kJ/mol)	R^2^
TSA	0.2	212.6	0.9639	0.2	214.4	0.9678
	0.3	223.6	0.9889	0.3	225.2	0.9900
	0.4	230.8	0.9928	0.4	232.3	0.9935
	0.5	241.3	0.9895	0.5	242.4	0.9905
	0.6	242.3	0.9923	0.6	243.6	0.9930
	0.7	228.5	0.9306	0.7	230.8	0.9381
	0.8	204.0	0.8665	0.8	209.0	0.8836
	0.9	347.8	0.9887	0.9	346.4	0.9897
	Average	241.4	-	Average	243.0	-
DSA	0.2	216.7	0.7886	0.2	218.0	0.8089
	0.3	229.9	0.8476	0.3	231.1	0.8623
	0.4	234.3	0.9157	0.4	235.5	0.9241
	0.5	238.0	0.9294	0.5	239.3	0.9365
	0.6	243.7	0.9034	0.6	245.0	0.9130
	0.7	201.0	0.4247	0.7	205.1	0.4807
	0.8	297.9	0.9576	0.8	299.1	0.9618
	0.9	311.2	0.9756	0.9	312.3	0.9780
	Average	246.6	-	Average	248.2	-
MSA	0.3	276.1	0.8929	0.3	274.7	0.9016
	0.4	274.2	0.9641	0.4	273.4	0.9673
	0.5	272.0	0.9926	0.5	271.5	0.9933
	0.6	271.5	0.9970	0.6	299.8	0.9767
	0.7	279.8	0.9987	0.7	279.4	0.9988
	0.8	327.0	0.9968	0.8	324.7	0.9971
	0.9	297.9	0.7843	0.9	299.5	0.8047
	Average	285.5	-	Average	289.0	-

**Table 3:** *E_a_* values for TSA based on the CR method.

Mechanism Functions	5 °C/min		10 °C/min		20 °C/min		Average Value	
	*E* (kJ/mol)	R^2^	*E* (kJ/mol)	R^2^	*E* (kJ/mol)	R^2^	*E* (kJ/mol)	R^2^
Reaction order model								
F0	75.1	0.9672	91.7	0.9928	78.1	0.9716	81.7	0.9716
F1	105.6	0.9910	121.3	0.9990	110.0	0.9913	112.3	0.9913
F2	32.2	0.8710	27.8	0.8518	33.9	0.8707	31.3	0.8707
F3	76.6	0.8719	64.4	0.8597	80.5	0.8733	73.9	0.8733
F4	130.8	0.8680	108.9	0.8529	137.5	0.8682	125.8	0.8682
Diffusional model								
1-D	163.6	0.9726	196.8	0.9938	170.1	0.9761	176.8	0.9761
2-D	181.5	0.9807	214.6	0.9965	188.8	0.9829	195.0	0.9829
3-D (J)	202.5	0.9876	234.9	0.9984	210.8	0.9887	216.1	0.9887
3-D (GB)	188.4	0.9834	221.4	0.9973	196.1	0.9851	202.0	0.9851
3-D (ZH)	248.3	0.9948	278.4	0.9990	258.7	0.9947	261.8	0.9947
Contracting geometry model								
R2	89.4	0.9821	105.7	0.9973	93.1	0.9839	96.1	0.9839
R3	94.6	0.9856	110.7	0.9981	98.5	0.9869	101.3	0.9869
Nucleation growth model								
A_1/2_	224.5	0.9921	256.0	0.9991	233.9	0.9924	238.1	0.9924
A_2/5_	284.0	0.9923	323.4	0.9991	295.8	0.9926	301.0	0.9926

**Table 4:** *E_a_* values for DSA based on the CR method.

Mechanism Functions	5 °C/min		10 °C/min		20 °C/min		Average Value	
	*E* (kJ/mol)	R^2^	*E* (kJ/mol)	R^2^	*E* (kJ/mol)	R^2^	*E* (kJ/mol)	R^2^
Reaction order model								
F0	44.1	0.9015	60.6	0.9948	45.5	0.9127	50.1	0.9363
F1	64.2	0.9518	81.2	0.9930	66.1	0.9586	70.5	0.9678
F2	16.8	0.8481	15.3	0.7023	17.2	0.8341	16.4	0.7949
F3	46.4	0.8840	40.6	0.7813	47.6	0.8738	44.9	0.8464
F4	82.6	0.8870	71.3	0.7899	84.7	0.8777	79.5	0.8515
Diffusional model								
1-D	101.6	0.9258	134.6	0.9958	104.8	0.9346	113.6	0.9521
2-D	113.3	0.9391	147.0	0.9961	116.9	0.9469	125.7	0.9607
3-D(J)	127.2	0.9517	161.1	0.9954	131.2	0.9583	139.8	0.9685
3-D(GB)	117.9	0.9438	151.7	0.9960	121.6	0.9512	130.4	0.9637
3-D(ZH)	157.4	0.9680	191.3	0.9915	162.3	0.9726	170.4	0.9774
Contracting geometry model								
R2	53.5	0.9313	70.4	0.9952	55.2	0.9402	59.7	0.9555
R3	56.9	0.9391	73.9	0.9947	58.7	0.9472	63.1	0.9603
Nucleation growth model								
A_1/2_	141.7	0.9609	175.8	0.9938	146.1	0.9665	154.5	0.9737
A_2/5_	180.5	0.9624	223.0	0.9940	186.1	0.9675	196.6	0.9747

**Table 5:** *E_a_* values for MSA based on the CR method.

Mechanism Functions	5 °C/min		10 °C/min		20 °C/min		Average Value	
	*E* (kJ/mol)	R^2^	*E* (kJ/mol)	R^2^	*E* (kJ/mol)	R^2^	*E* (kJ/mol)	R^2^
Reaction order model								
F0	45.0	0.9919	49.5	0.9941	46.8	0.9930	47.2	0.9931
F1	69.4	0.9753	76.1	0.9806	72.2	0.9763	72.7	0.9775
F2	24.6	0.7253	27.7	0.7563	25.6	0.7263	26.0	0.7364
F3	63.0	0.7753	69.5	0.7941	65.6	0.7761	66.1	0.7820
F4	110.5	0.7885	121.1	0.8043	114.9	0.7892	115.6	0.7941
Diffusional model								
1-D	103.0	0.9936	112.4	0.9953	107.2	0.9945	107.7	0.9945
2-D	117.2	0.9902	127.8	0.9928	121.9	0.9911	122.5	0.9914
3-D(J)	134.1	0.9849	146.2	0.9884	139.5	0.9858	140.1	0.9864
3-D(GB)	122.8	0.9885	133.9	0.9914	127.8	0.9894	128.3	0.9898
3-D(ZH)	171.3	0.9723	186.6	0.9774	178.2	0.9733	178.9	0.9743
Contracting geometry model								
R2	56.3	0.9849	61.9	0.9888	58.6	0.9860	59.0	0.9866
R3	60.5	0.9819	66.4	0.9863	63.0	0.9830	63.4	0.9838
Nucleation growth model								
A_1/2_	152.3	0.9712	165.9	0.9834	158.3	0.9755	158.8	0.9679
A_2/5_	193.7	0.9698	210.8	0.9817	201.3	0.9766	201.9	0.9765

**Table 6:** The IR characteristic absorption bands of major products and functional groups.

Products	Wavenumbers (cm^−1^)	Functional Groups
H_2_O	3500–4000; 1500–1700	O-H
C_2_H_4_	951 and 3099	=C–H/C=C
CH_4_	1303 and 3010	C-H
CO_2_	2350–2400; 660–700	O=C=O

**Table 7:** Thermodynamic parameters of HSAs at different degrees of conversion.

Samples	α	*lnA* (s^−1^)	Δ*H* (kJ/mol)	Δ*S* (J·mol^−1^·K^−1^)	Δ*G* (kJ/mol)
TSA	0.2	47.63	207.91	134.15	135.47
	0.3	50.09	218.58	154.31	135.25
	0.4	51.70	225.55	167.47	135.11
	0.5	53.99	235.57	186.40	134.92
	0.6	54.26	236.64	188.40	134.90
	0.7	51.35	223.63	163.88	135.14
	0.8	46.40	201.02	121.18	135.59
	0.9	77.52	338.16	379.33	133.32
DSA	0.2	47.99	211.67	137.42	136.77
	0.3	50.93	224.47	161.39	136.51
	0.4	51.93	228.76	169.42	136.43
	0.5	52.78	232.43	176.29	136.35
	0.6	54.07	237.99	186.69	136.25
	0.7	45.07	197.61	111.12	137.05
	0.8	66.20	290.74	285.13	135.34
	0.9	69.16	303.67	309.21	135.15
MSA	0.3	60.27	268.27	239.43	136.82
	0.4	59.98	266.72	236.57	136.85
	0.5	59.56	264.73	232.89	136.88
	0.6	65.87	292.92	285.06	136.42
	0.7	61.33	272.42	247.13	136.75
	0.8	71.40	317.49	330.47	136.06
	0.9	65.80	290.92	281.40	136.43

**Table 8:** The pyrolysis mechanism functions commonly used.

Mechanism Functions	*f*(*α*)	*g*(*α*)
Reaction order model		
Zero-order (F0)	1	α
First-order (F1)	(1 − *α*)^1^	−ln(1 −*α*)
Second-order (F2)	(1 − *α*)^2^	(1 − *α*)^−1^ − 1
Third-order (F3)	(1 − *α*)^3^	[(1 − *α*)^−2^ − 1]/2
nth-order (Fn)	(1 − *α*)^n^	[(1 − *α*)^1 − n^ − 1]/(n − 1)
Diffusional model		
1-D	1/2*α*	*α* ^2^
2-D	1/[−ln(1 − *α*)]	(1 − *α*)ln(1 − *α*)+*α*
3-D (J)	3(1 − *α*)^1/3^/2[(1 − *α*)^−1/3^ − 1]	[1 − (1 − *α*)^1/3^]^2^
3-D (GB)	3/[2((1 − *α*)^−1/3^ − 1)]	(1 − 2*α*/3) − (1 − *α*)^2/3^
3-D (ZH)	1.5(1 − *α*)^4/3^[(1 − *α*)^−1/3^ − 1]^−1^	[(1 − *α*)^−1/3^ − 1]^2^
Contracting geometry model		
Contracting area (R2)	2(1 − *α*)^1/2^	1 − (1 − *α*)^1/2^
Contracting volume (R3)	3(1 − *α*)^2/3^	1 − (1 − *α*)^1/3^
Nucleation growth model		
A_1/2_	[(1 − *α*)[−ln(1 − *α*)]^−1^]/2	[−ln(1 − *α*)]^2^
A_2/5_	2[(1 − *α*)[−ln(1 − *α*)]^−(3/2)^]/5	[−ln(1 − *α*)]^5/2^

## Data Availability

The data presented in this study are available on request from the corresponding author.
